# The Effect of the Open and Closed System Suctions on Cardiopulmonary Parameters: Time and Costs in Patients Under Mechanical Ventilation

**Published:** 2014-06-15

**Authors:** Ali Afshari, Mahmoud Safari, Khodayar Oshvandi, Ali Reza Soltanian

**Affiliations:** 1 Department of Medical-Surgical Nursing, Faculty of Nursing and Midwifery, Hamadan University of Medical Sciences, Hamadan, IR Iran; 2 Research Center for Maternal and Child Care, Hamadan University of Medical Sciences, Hamadan, IR Iran; 3 Department of Biostatistics and Epidemiology, Faculty of Health, Hamadan University of Medical Sciences, Hamadan, IR Iran

**Keywords:** Suction, Respiration, Artificial, Nursing, Iran

## Abstract

**Background:**

One of the measures to keep the airway open is suctioning of endotracheal tube in patients under ventilation. This procedure can be accompanied with some complications. Selection of appropriate method of suctioning can prevent incidence of acute complications.

**Objectives:**

This study aimed to compare the effects of the open and closed system suctioning methods on blood pressure, mean arterial pressure, heart rate, percentage of arterial oxygen saturation, time, and costs in patients under mechanical ventilation.

**Patients and Methods:**

This clinical trial study was conducted on 40 patients in ICU. Patients’ blood pressure, heart rate, arterial oxygen saturation, related costs, and length of suctioning procedure were measured and recorded immediately before and one, five, ten, and fifteen minutes after suctioning. Data were analyzed using paired t test and repeated measure analysis of variance.

**Results:**

No significant differences were observed between the two suctioning methods in terms of mean systolic blood pressure (P = 0.075), diastolic blood pressure (P = 0.405), and mean arterial pressure (P = 0.257) in the five consecutive measurements. However, significant changes were observed in heart rate (P = 0.025) and percentage of arterial oxygen saturation (P < 0.001). The mean lengths of time in open and closed suctioning methods were 5.59 ± 0.211 and 4.34 ± 0.039 seconds, respectively (P < 0.001). The cost of the closed system was lower than the open method for the patients who were admitted to ICU for longer than two days.

**Conclusions:**

Closed suction caused fewer disturbances in patients’ hemodynamic condition, took shorter time, and is more economical. Therefore, this method can replace open suction method in caring of severely critically ill patients.

## 1. Background

The aim of endotracheal tube (ETT) suctioning in patients under mechanical ventilation is keeping the airways open through removal of accumulated pulmonary secretions (1). This procedure is administrated eight to 17 times a day for each patient under mechanical ventilation (2). Although ETT suctioning is a vital procedure, it may results in complications such as discomfort, infection, bleeding, tracheal mucosal injury, increase in intracranial pressure, atelectasis, cardiac dysrhythmia, and hemodynamic changes in patients (3, 4). Despite the evidence-based protocols and instructions, low knowledge of nurses about ETT suctioning is considered as the main cause for most of these complications (5).

Nowadays two methods are used for ETT suctioning. The most common method in Iran is open system suctioning method, which need participation of two nurses and may lead to temporary disruption of ventilation and oxygen supply due to disconnection of the patient from ventilation device during suctioning (6). The most important risk factor in open method of ETT suctioning is hypoxia (7). However, in the second method, which is known as closed suction system, ETT suctioning can be administrated through connections in closed suction set and while the ventilation is performing without disconnecting the patient from ventilator (8).

In recent years, several studies have compared the effects of the two suctioning methods (9-14); however, the results are contradictory. For example, changes in heart rate were significant in one study (9), which was in contrary to findings of the other investigations (10, 11). In addition, changes in arterial oxygen saturation between two procedures were significant in two studies (11, 12), while they were not significant in a study reported by Fernandez et al. (10). Changes in mean arterial pressure were significant between the two procedures in a study by Lee et al. (9), but they were not significant in the other studies (10, 11). Conflicting results were also reported in terms of the costs of the closed versus open suctioning system (13, 14). In most of these studies, researchers suggested further investigations in this field in order to get more reliable results. 

## 2. Objectives

The present study aimed to compare the effect of the open and closed system suctioning methods on cardiorespiratory parameters (i.e. blood pressure, mean arterial pressure, heart rate, and percentage of arterial oxygen saturation), time, and costs in patients under mechanical ventilation. 

## 3. Patients and Methods

This clinical trial was conducted during May through February 2011 in intensive care unit (ICU) of Be’sat Hospital in Hamadan, Iran. Study population included all patients with an ETT. The sample size was calculated based on the standard deviation of systolic blood pressure before and after suction in similar studies (15). The least significant difference between the mean values were considered to be equal to five units. The type 1 error was also selected as 0.05 and the test power as 0.90. Then, 38 patients were estimated to be recruited in the study; however, we recruited 40 patients. Inclusion criteria were as follows: participants’ age of 18 to 65 years; having a ETT and being connected to ventilator; ventilation with SIMV mode, PEEP = 5 cm H_2_O, and FIO_2_ = 50%; having stable hemodynamic parameters (blood pressure, mean arterial pressure, and heart rate); and no intake of inotropic medications. Exclusion criteria were instability in hemodynamic parameters, administration of emergency diagnostic or treatment interventions for the patient during the study, changes in ventilator settings, and the need to resuctioning between administering of the open and closed suctioning. 

The subjects were selected by purposive sampling and both methods of ETT suctioning performed for each patient (open-closed or closed-open). Suctioning methods were administrated based on the protocol of American Association for Respiratory Care (AARC). In this protocol, all standards of interventions before, during, and after ETT suctioning methods have been presented in the form of a checklist (16). Before ETT suctioning, the patients became hyperoxygenated for two minutes by 100% oxygen and ETT suctioning was performed one time (ten second) using 120 mm Hg negative pressure. Both methods of ETT suctioning were performed (open-closed or closed-open) in a random manner for each patient with 90 minutes interval. All patients became hyperoxygenated using 100% oxygen for two minutes after the suctioning procedures.

Blood pressure, mean arterial pressure, heart rate, and percentage of arterial oxygen saturation were recorded immediately before and one, five, ten, and fifteen minutes after suctioning using a cardiac monitor (cardio set LX 110 Sa Iran, Iran). The time spent on ETT suctioning (from tuning suction negative pressure up to the end of suctioning procedure) for each patient was recorded in both methods using a digital timer (ULTRAK 330 digital timer, China). 

The costs of the used items and equipment related to each suctioning method were calculated while personnel’s cost was not calculated due to dissimilarity of income and salary of nursing staff. A special form was assigned to each patient to record the items used in ETT suctioning and any other item used. In addition, the amount of normal saline, which was used to rinse suctioning catheter, were recorded. Then the cost of each suctioning method was calculated. 

In open system, almost all items including gloves and suction catheter were disposable and only the suction connection was changed each 48 hours. The costs were calculated considering the frequency each item was used. Closed suction set is reusable (17) and its cost was calculated considering the times of changing the close suction set and its related connections. In the next step, the costs of used items in both suctioning methods were calculated with regard to their length of consumption and number of changes as well as the duration of hospitalization in intensive ICU. 

### 3.1. Ethical Considerations

The Research Council and the Human Research Ethics Committee of Hamadan University of Medical Sciences approved the study protocol and its ethical considerations. To begin the study, the researcher explained the study process to the patients’ family and they signed a written informed consent. The patients’ family were also assured about data confidentiality, safeness of the study, and their right of not to participate. We also observed all ethical issues in accordance with the last version of the Helsinki Declaration. 

### 3.2. Data Analysis

SPSS 16 (SPSS Inc., Chicago, IL, USA) was used to analyze the data. Paired t test and repeated measure analysis of variance (ANOVA) were used to compare variables between the two methods. In addition, descriptive statistics were employed for demographic variables. A P value less than 0.05 was considered as statistically significant. 

## 4. Results

Most of the patients were male (72.5%) with the mean age of 42.15 years. The majority of patients were hospitalized in ICU due to neurologic injuries (40%) (Table 1). A significant difference was observed between the mean of heart rate immediately before and at one, five, ten, and fifteen minutes after suctioning through the open and closed system methods. Heart rate significantly increased after the open suction in comparison to the closed system (P = 0.025). A significant difference was observed in arterial O_2_ saturation between the open and closed methods immediately and at the five consecutive measurements (P < 0.001). Percentage of arterial O_2_ saturation in open suctioning method showed a more reduction in comparison to the closed system. However, repeated measure ANOVA showed no significant differences between the two suctioning methods in terms of mean systolic blood pressure (p = 0.075), diastolic blood pressure (P = 0.405), and mean arterial pressure (P = 0.257) in the five consecutive measurements (Table 2). The mean lengths of time in the open and closed suctioning methods were 5.59 ± 0.211 and 4.34 ± 0.039 minute, respectively. Paired t test showed a significant difference in the mean lengths of time between the two suctioning methods (P < 0.001) (Table 3).

**Figure 1.**
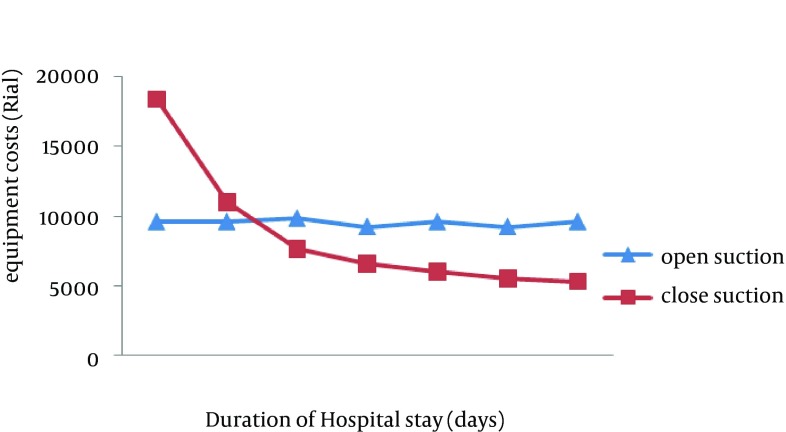
Comparison of Consumed Items Costs Between Open and Closed Suction

In the present study, the used items and their final costs were estimated and recorded for both the open and closed suctioning methods. The final cost for each open system suctioning was 9572 Rials (equal to 30 cents), while it was 18 403 Rials (around 60 cents) for the first attempt in the closed system (almost two folds more). However, with regard to the frequent usage of a closed system set and mean length of patients’ hospitalization in ICU, the final cost of the closed system would be lower than the open method for the patients who were hospitalized in ICU for more than two days (Figure 1).

**Table 1.** Basic Characteristics of the Patients

Variable	No. (%)
**Sex**	
Male	29 (72.5)
Female	11 (27.5)
**Age, y**	
18-39	16 (40)
40-65	24 (60)
**Hospitalization reason**	
Neurological injurers	16 (40)
Respiratory problems	14 (35)
Postoperative cares	10 (25)

**Table 2.** Cardiopulmonary Variables Before and After Suctioning in Patients Under Mechanical Ventilation ^a^

Variable	Before	1 min after	5 min after	10 min after	15 min after	P Value
**Systolic blood pressure**						0.075
Open	119.9 ± 9.1	125 ± 12.3	125.4 ± 8.1	120.2 ± 9.4	119.2 ± 9.3	
Closed	117.5 ± 9.2	121.2 ± 9.9	119.8 ± 10.1	112.4 ± 9.2	117.4 ± 9.7	
Paired t test	t = 2.99, P = 0.005	t = 6.27, P = 0.001	t = 5.35, P = 0.001	t = 2.05, P = 0.046	t = 1.92, P = 0.061	
**Diastolic blood pressure**						0.405
Open	71.1 ± 6.5	75.1 ± 7.1	73.6 ± 6.3	71.6 ± 6.3	71.3 ± 5.7	
Closed	70.8 ± 7.4	72.9 ± 6.7	71.5 ± 8.4	70.3 ± 7.8	71.3 ± 6.2	
Paired t test	t = 0.31, P = 0.753	t = 2.95, P = 0.005	t = 2.15, P = 0.038	t = 1.51, P = 0.139	t = 0.154, P = 0.878	
**Mean arterial pressure**						0.287
Open	82.6 ± 6.6	85.9 ± 8.0	83.5 ± 8.6	82.3 ± 8.3	81.8 ± 7.6	
Closed	81.1 ± 8.7	82.2 ± 8.2	82.5 ± 8.9	81.0 ± 8.9	80.9 ± 8.3	
Paired t test	t = 1.84, P = 0.072	t = 4.23 P < 0.001	t = 2.74, P = 0.009	t = 1.85, P = 0.071	t = 1.47, P = 0.149	
**Heart rate**						0.025
Open	84.8 ± 9.8	95.3 ± 11.1	91.2 ± 11.0	87.0 ± 10.4	85.2 ± 9.8	
Closed	84.0 ± 9.6	87.5 ± 10.3	85.3 ± 10.4	82.9 ± 9.9	83.6 ± 9.4	
Paired t test	t = 1.38, P = 0.175	t = 12.21, P < 0.001	t = 8.72, P < 0.001	t = 6.75, P < 0.001	t = 2.79, P = 0.008	
**Arterial O_2_** ** saturation**						0.001
Open	97.7 ± 1.4	93.2 ± 1.9	95.2 ± 1.7	96.5 ± 1.9	96.7 ± 1.5	
Closed	98.0 ± 1.4	97.3 ± 1.9	97.0 ± 1.5	97.2 ± 1.4	97.4 ± 1.4	
Paired t test	t = -2.39, P = 0.022	t = -13.5, P < 0.001	t = -8.54, P < 0.001	t = -2.94, P = 0.005	t = -3.47, P = 0.001	

^a^ Data are presented as mean ± SD.

**Table 3.** Comparison of the Length of Suctioning With Open and Closed System Methods

Type of suction	mean ± SD, min	Paired t test
**Open**	5.59 ± 0.211	t = 35.58, df = 39, P < 0.001
**Closed **	4.34 ± 0.039	

## 5. Discussion

The main goal of the present study was to compare the changes in arterial O_2_ saturation, heart rate, blood pressure, and costs as well as time spent in the patients under mechanical ventilation after ETT suctioning through the open and closed methods. The results of this study showed more reductions in arterial O_2_ saturation in using the open system in comparison to the closed suctioning method. Such a reduction in arterial O_2_ saturation in patients under mechanical ventilation may lead to hypoxia during the open system suctioning. Several previous studies confirmed that using the open suctioning system was accompanied with the risk of hypoxia in patients. This problem occurs because not only the patient is disconnected from the ventilator during suctioning, but also the existing air in patents’ lungs is sucked out by the suction device (18-22). However, the risk of hypoxia is lower in using the closed suctioning system because patients are not disconnected of the ventilator and ventilation is continued while suctioning system is running (18).

The present study showed a significant difference between the patients’ heart rate in the two suctioning methods. Although the postsuctioning heart rate was variant in the both methods, changes were more observable in the open suctioning method especially at first and fifth minutes after suctioning. This finding is consistent with the report of Lindgren et al. who studied the effectiveness and side effects of the closed and open suctioning methods (23). Others studies have also showed that patients’ heart rate was significantly increased in the open suctioning method comparison to the closed technique (11, 13, 18). Such changes are related to hypoxia induced by open suctioning method. Then, hypoxia stimulates the adrenergic nervous system, which is responsible for cardiovascular and hemodynamic responses such as tachycardia, as a compensatory response to the decrease in blood O_2_ saturation (24, 25). Moreover, heart rate may be affected by the anxiety in patients under mechanical ventilation (26); attendance of two nurses in the open suction procedure, in comparison to one nurse in the closed system, might lead to patients’ anxiety (27).

The current study showed that the patients’ systolic and diastolic blood pressures were not significantly different after the two suctioning methods. This finding is consistent with that of Jongerden et al. (28). However, it is in contrast to the result of Lee et al. (9). Airways suctioning is an invasive procedure and can result in increased blood pressure as well as heart rate through sympathetic system stimulation mechanisms (29). It can be noted that in similar studies, the selected subjects had various modes of ventilation and the patients did not have similar PEEP while in the present study, all the subjects were under SIMV mode and their PEEP was fixed as 5 cm H_2_O in ventilator. 

With regard to 85 seconds difference in the mean length of the open and closed suctioning methods, the number of active beds in the related ward (23 beds), and the mean number of ETT suctioning (12 times in 24 hours), the mean difference in time of the two methods would be about six and half hours a day, 195.5 hours a month, and 2346 hours a year. These working hours are equal to working hours of a nurse in a year. Therefore, it can be concluded that application of the close suctioning method in ICU not only could be safer for patient, but also would improve the nurse productivity. 

The present study showed that the cost of the closed system suction in ICU was higher for patients with a duration of ICU stay for less than 48 hours; however, the cost would be decreased significantly for patients with a duration of ICU stay of more than 48 hours. The costs would also be lower for patients who need ETT suctioning for more than 14 times a day. In some studies, it was reported that the costs of the closed suction was more than the open method (13, 30). However, the findings of the present study were consistent with studies by Dodek et al. and Lorente et al. who reported that the costs of the closed suction system are lower than the open one, especially in patients with hospitalization for more than four days (14, 17). The lower costs of the closed suction system could be attributed to the possibility for longer use of the suction set and needing to fewer staff for suctioning procedure (14, 17). 

The results of this study showed that use of the closed suctioning system causes fewer hemodynamic disturbances in patients under mechanical ventilation. It also is more economical in terms of time and costs for patients and the health care system. Therefore, closed system suctioning can be an appropriate method for patients’ ETT suctioning and is recommended to be used in other ICUs. There was not any limitation in this study because of attending of one group of patients in the two suctioning methods (open and closed). Finally, investigating of the effects of suctioning methods on arterial blood gas and the incidence of ventilator-associated pneumonia are recommended.
